# The frequency of *DRD2* rs1076560 and *OPRM1* rs1799971 in substance use disorder patients from the United Arab Emirates

**DOI:** 10.1186/s12991-018-0192-4

**Published:** 2018-06-01

**Authors:** Hiba Alblooshi, Gary Hulse, Wael Osman, Ahmed El Kashef, Mansour Shawky, Hamad Al Ghaferi, Habiba Al Safar, Guan K. Tay

**Affiliations:** 1 School of Human Sciences, The University of Western Australia, Crawley, WA Australia; 2 School of Psychiatry and Clinical Neurosciences, The University of Western Australia, Crawley, WA Australia; 3 School of Medical and Health Sciences, Edith Cowan University, Perth, WA Australia; 4 Center of Biotechnology, Khalifa University of Science, Technology and Research, PO Box 1227788, Abu Dhabi, United Arab Emirates; 5 United Arab Emirates National Rehabilitation Center, Abu Dhabi, United Arab Emirates; 6 Faculty of Biomedical Engineering, Khalifa University of Science, Technology and Research, Abu Dhabi, United Arab Emirates

**Keywords:** Substance use disorder, *DRD2* gene, *OPRM1* gene, rs1076560, rs1799971, UAE

## Abstract

**Background:**

Dopaminergic and opioid systems are involved in mediating drug reward and reinforcement of various types of substances including psychoactive compounds. Genes of both systems have been candidate for investigation for associations with substance use disorder (SUD) in various populations. This study is the first study to determine the allele frequency and the genetic association of the *DRD2* rs1076560 SNP and *OPRM1* rs1799971 SNP variants in clinically diagnosed patients with SUD from the United Arab Emirates (UAE).

**Methods:**

A cross-sectional case–control cohort that consisted of 512 male subjects was studied. Two hundred and fifty patients with SUD receiving treatment at the UAE National Rehabilitation Center were compared to 262 controls with no prior history of mental health and SUD. DNA from each subject was extracted and genotyped using the TaqMan^**®**^ SNP genotyping assay.

**Results:**

There were no significant associations observed for *DRD2* rs1076560 SNP, *OPRM1* rs1799971 SNP, and combined genotypes of both SNPs in the SUD group.

**Conclusion:**

Further research is required with refinements to the criteria of the clinical phenotypes. Genetic studies have to be expanded to include other variants of the gene, the interaction with other genes, and possible epigenetic relationships.

## Background

The dopaminergic and opioid systems are part of a network involved in rewarding response following the consumption of opioids and other psychoactive substances [1, 2]. The dopamine system has been central to theories in reward of substance use disorder (SUD) that has been debated for several decades [3]. The consumption of addictive substances stimulates the release of dopamine into nucleus accumbens (NAc) elevating the dopamine level to above basal levels [4]. There are different mechanisms of action and target molecules in the dopamine system for the variety of substances that are commonly consumed. This dopaminergic system comprises an array of dopamine receptors, transporters, and substance-metabolising enzymes. Members of the family of the dopamine receptor genes, *DRD1*, *DRD2*, *DRD3*, *DRD4,* and *DRD5*, have been widely studied as risk factors for SUD [2]. The dopamine D2 receptor is a part of G protein-coupled receptors (GPCRs) that is encoded by the *DRD2* gene. It is located on chromosome 11q23, spanning a region of 65.56 kilobases and comprises 8 exons separated by 7 introns. During the splicing process of the *DRD2* mRNA precursor, two alternative subtypes of the D2 receptors are formed: a 443 amino acid D2L or a 414 amino acid D2S form. The longer D2L form is more common [5]. This 29 amino acid difference between the two isoforms does not appear to affect the pharmacological properties of the dopamine D2 receptor. The D2L/S variation changes the localization of the third intracellular loop of the receptor that interacts with the G coupled protein; hence, it affects the intracellular signalling mechanism. The mechanisms of dopamine receptor signal transduction and regulation are not only mediated via G protein signalling, but also involve G protein independent signalling events [6].

Understanding the reward and the treatment responses highlight the necessity of reviewing the relation between the genetic variants of these dopaminergic genes and SUD [7]. Patriquin et al. [7] reviewed the correlation of dopaminergic genes to SUD. The genetic variants of *DRD2* have been a focus of intense research to determine their link to SUD. Two single-nucleotide polymorphisms (SNPs) of the *DRD2* loci; rs2283265 in intron 5 and rs1076560 in intron 6 have been reported to be associated with cocaine use [8]. A *DRD2* variant (rs1076560) has also been studied in various populations. Clark et al. [9] reported the association between rs1076560 and opioid use in African Americans (AA) (*p *= *0.03*) and European Americans (EA) (*p *= *0.02*). These findings introduced insights into the possible roles of these dopaminergic variants on SUD. However, the extent of genetic variations acting as a risk factor for SUD is still not understood.

The opioid receptor gene family has been extensively studied to identify if there are any associations with SUD. There three subtypes are μ, κ, and δ, encoded by the *OPRM1*, *OPRK1*, and *OPRD1* genes, respectively. The product of the *OPRM1* gene plays a role in facilitating the analgesia and euphoria effects of opioids. The G protein-coupled mu opioid receptor encoded by the *OPRM1* gene is a multiple trans-membrane protein that has a high affinity for endogenous and exogenous opioids [10]. The *OPRM1* gene consists of 9 exons which encode over 100 variants that produce between 19 and 39 splice forms of the protein [11]. The rs1799971 (A118G) site is an SNP that is located in exon 1 of the *OPRM1* gene. This variant encodes a missense change in *OPRM1* at position 40 resulting in a change from an asparagine to an aspartate (Asn40Asp) in the extracellular domain of the receptor. This substitution eliminates an *N*-glycosylation site in the extracellular domain, which affects endogenous opioid binding and receptor activity [12]. The role of the rs1799971 in SUD remains in dispute [13, 14]. The effect of the variants of rs1799971 on different classes of substance has been extensively studied in various populations [10, 11, 13, 15–20]. However, only two studies [21, 22] have looked into rs1799971 variants in the Arab population. Several studies have reported associations between A118G with different substances of use in patients from different ethnic groups [10, 11, 13, 15–20], with others not finding significant associations with SUD [23, 24].

This study is the first study to report on the allele frequency for the rs1076560 SNP of *DRD2* and the rs1799971 SNP of *OPRM1* in individuals with SUD from the United Arab Emirates (UAE). This case–control study investigated the genetic association between the *DRD2* rs1076560 SNP and *OPRM1* rs1799971 SNP and SUD in the UAE population. The allele frequencies of *DRD2* rs1076560 SNP in the UAE population were compared to other global populations. The allele frequencies of the *OPRM1* rs1799971 SNP in this UAE study were compared with SUD cohorts in other Arab populations.

## Methods

### Subject information

A total of 250 male nationals of the UAE were recruited from the National Rehabilitation Center (NRC) based on the nation’s capital of Abu Dhabi. All participants were previously diagnosed with SUD based on the DSM-5 criteria. Saliva samples were collected from each patient who had agreed to participate in this study, using the DNA Oragene saliva kit (DNA Genotek, Ottawa, Ontario, Canada). In addition, 262 male nationals of the UAE with no prior history of SUD or mental illness were recruited as controls. These individuals were part of an ongoing population study towards the establishment of the Emirates Family Registry (EFR) [25]. The characteristics of the cohort are summarised in Alblooshi et al. [26] which includes socio-demographic data as well as the combination and types of substances that were used. The study was conducted in accordance with standards set by the World Medical Association of Helsinki [27]. Specifically, approval to study human subjects was obtained from the NRC in Abu Dhabi. In addition, reciprocal approval was obtained from the human ethic committee at the University of Western Australia (RA/4/1/6715).

### Genotyping of single-nucleotide polymorphism (SNP)

Genomic DNA was isolated from the cells in human saliva samples using the laboratory protocol for manual extraction of DNA as recommended by Genoteck (Ottawa, Ontario, Canada). SNP genotyping was preformed using a TaqMan^®^ SNP genotyping assay on the viiA™7 (Applied Biosystems Inc. (ABI); Foster City, CA, USA). For quality control (QC) purposes, 10% of samples that were studied were randomly selected. These QC samples were genotyped at least twice. There was 100% concordance between the genotypes recorded for replicates from the same individual. The Hardy–Weinberg equilibrium (HWE) was calculated for both the cases and controls. No significant deviation from HWE was observed.

### Identification and inclusion criteria of relevant studies for comparison purposes

Life science journal articles containing information related to genotyping studies of the two SNPs of interest: *DRD2* rs1076560 and *OPRM1* rs1799971 were retrieved from a search of electronic publication databases. Specifically, articles in PubMed/MEDLINE (US National Library of Medicine), EMBASE (Elsevier B.V., Amsterdam, The Netherlands), and ISI Web of Science (Thomson Reuters, New York, NY, USA) that were published to 15 March 2017 were retrieved. The search process was set up to specifically identify case–control studies that examined associations between each SNP (*DRD2* rs1076560 and *OPRM1* rs1799971) with different types of SUD in different populations or ethnic groups. Data that were specifically extracted from these published studies for comparison included: (1) the number of cases and controls; (2) the ethnicity of the study population; (3) the genotyping method used; (4) allele and genotype frequency data; (5) information related to Hardy–Weinberg equilibrium; and (6) the significance of the levels of associations identified (*p* values and statistical tests).

### Statistical analysis

Allele and genotype frequencies in the cases and controls from this study were calculated using the GenAlex package (Peakall and Smouse 2006, 2012) and association was determined using the STATA statistical software (College Station, TX, USA).

## Results

The allele frequencies of the *DRD2* rs1076560 SNP in the patient and control groups were compared. The Minor Allele Frequency (MAF) for *DRD2* rs1076560 was the “A” allele, with a frequency of 11.80% in the substance use group compared with 13.20% in the controls. Correspondingly, the “C” allele was 88.20% in cases and 86.80% in controls (Table 1). The *χ*^2^ allelic association between the *DRD2* rs1076560 SNP and substance use was not significant in the UAE population that was studied [*p *= 0.52, odds ratio (OR) = 0.88].

**Table 1:** Summary of the meta-analysis of the *DRD2* rs1076560 in association with SUD in different populations

Population	Substance	Phenotype	Number	Allele’s frequency (%)		*p*	OR (95% CI)	References
				C	A			
Caucasians	Alcohol	Case	171	79.00	21.00	0.14	1.34 (0.90–1.98)	[30]
		Control	160	83.00	17.00			
African Americans	Opioid	Case	278	88.00	12.00	*0.03*	1.43 (1.04–1.97)	[9]
		Control	750	91.00	9.00			
European Americans		Case	999	83.00	17.00	*0.02*	1.27 (1.04–1.54)	
		Control	656	86.00	14.00			
African Americans	Cocaine	Case	45	94.00	6.00	0.53	0.66 (0.18–2.40)	[27]
		Control	31	92.00	8.00			
Caucasians		Case	74	76.00	24.00	*0.003*	2.74 (1.38–5.45)	
		Control	63	90.00	10.00			
Japanese	Alcohol	Case	297	59.90	40.10	*0.03*	1.30 (1.02–1.66)	[29]
		Control	425	66.00	34.00			
Jordanian Arabs	Poly-substance	Case	220	84.30	15.70	*0.03*	1.53 (0.90–2.68)	[30]
		Control	240	89.20	10.80			
UAE Cohort	Mixed^a^	Case	250	88.20	11.80	0.52**	0.88 (0.61–1.28)	This study
		Control	262	86.80	13.20			

*CI* confidence intervals, *OR* odds ratio

** *p* value of Armitage test using the status of mixed opioids (*n* = 250) versus no addiction (*n* = 262)

^a^Mixed: include single substance and poly-substance users

The results of this UAE study were compared with published data that included association studies between the *DRD2* rs1076560 SNP and the use of different substances (e.g., alcohol, cocaine, opioids, and poly-substances) in a number of different populations (e.g., Caucasians, African Americans, Asians, and Jordanian Arabs) (Table 1). Six relevant publications matched the selection criteria described in the “Methods” section. All the studies identified were case versus control studies. Clark et al. [9] studied a relatively large population of EA and AA (999 EA cases versus 656 EA controls and 278 AA cases versus 750 AA controls) and showed that the *DRD2* rs1076560 SNP was significantly associated with opioid use in both populations with *p* values of 0.02 and 0.03, respectively. The MAF of the *DRD2* rs1076560 SNP was higher in EA case group (17.0%) when compared to EA control group (14.0%) as well as in AA cases (12.0%) versus AA controls (9.0%) [9]. Moyer et al. [28] studied cocaine users in the same two ethnic groups (EA and AA) and showed that the *DRD2* rs1076560 SNP was associated with cocaine use in EA (*p* = 0.003, OR = 2.74), but not in AA (*p* = 0.53, OR = 0.66). A polish study of European alcohol users reported results that were not significant (*p *= 0.14, OR = 1.34). In a study of Japanese patients, the risk allele “A” of the *DRD2* rs1076560 SNP was associated with alcohol use (*p* = 0.03, OR = 1.30) [29]. To date, there has only been one other study of patients of Middle Eastern descent. Al-Eitan et al. [30] found the *DRD2* rs1076560 SNP to be associated with poly-substance use in a Jordanian Arab population (*p* = 0.03, OR = 1.53).

The *OPRM1* rs1799971 SNP genotype frequencies studied in two case–control studies of SUD in populations of Arab descent are summarised in Table 2. In this study, the MAF “G allele” was 15.4% in cases and 18.9% in controls. Overall, the association of the *OPRM1* rs1799971 SNP was not significant in the UAE patients with SUD (*p* = 0.12, OR = 0.78). In comparison with an Egyptian Arab population, no significant association was reported between the *OPRM1* rs1799971 SNP and Tramadol use (*p* = 0.54, OR = 0.73) with MAF “G allele” of 5.2% in cases and 7.0% in controls. Simple combinations in both populations indicate significant association between the MAF “G allele” with SUD (*p* = 0.04, OR = 0.73). This enhanced the odd ratio value with no heterogeneity observed. The combined data were adjusted using Cochran–Mantel–Haenszel test that was close to the UAE cohort.

**Table 2:** Distribution of the allele frequency of rs1799971 among Arab population

Cohort	Case					Control					p*	OR (95% CI)	*p-*hetero**
	AA	AG	GG	Sum	MAF (%)	AA	AG	GG	Sum	MAF (%)			
UAE	175	73	2	250	15.4	171	83	8	262	18.9	0.12	0.78 (0.56–1.08)	
Egypt-Arabs	69	8	0	77	5.2	43	7	0	50	7.0	0.54	0.73 (0.26–2.07)	
Simple combination	244	81	2	327	13.0	214	90	8	312	17.0	*0.04*	0.73 (0.51–0.99)	
M-H adjusted^a^												0.78 (0.57–1.06)	0.75

*CI* confidence intervals, *MAF* minor allele frequency, *OR* odds ratio

* *p* value of Cochran–Armitage test using allelic model

** *p*-hetero: *p* value of heterogeneity of Breslow–Day of homogeneity test

^a^Cochran–Mantel–Haenszel test (CMH) is a test used in the analysis of stratified or matched categorical data

The combined genotype frequencies for the *DRD2* rs1076560 SNP and *OPRM1* rs1799971 SNP are summarised in Table 3. There were no significant associations between the combined genotypes of both SNPs in cases (*p* = 0.88) and controls (*p* = 0.23). The combined genotype CC/AA was the highest in cases (55.6%) and controls (50.8%). This was followed by the combined genotype CC/AG with similar representation in cases (22.0%) and controls (22.9%). The combined genotype of the AC/GG was not observed in any individuals in the case group. Whereas, this combined genotype was observed in 1.2% of the control group. There were no cases or controls subjects with the combined genotype, AA/GG.

**Table 3:** *DRD2* rs1075650 and *OPRM1* rs1799971 genotype combination among case–control of this cohort

*SNPs*		*OPRM1* rs1799971 Genotype							
		Case			*p*	Control			*p*
		AA	AG	GG		AA	AG	GG	
*DRD2 rs1076560 genotype*									
CC		55.60	22.00	0.80	0.88	50.76	22.90	1.91	0.23
AC		13.20	6.40	0.00		12.60	8.78	1.15	
AA		1.20	0.80	0.00		1.91	0.00	0.00	

## Discussion

In the UAE cohort represented in this study, there was no significant genetic association between the *DRD2* rs1076560 SNP (*p* = 0.52) and SUD. The MAF of the *DRD2* rs1076560 SNP was higher in the controls (13.2%) when compared to the substance users (11.8%). This was similar to the observations made in an AA population studied by Moyer et al. [28], where the MAF in cocaine users (6.0%) was lower than in the control group at 8.0%. In general, Moyer et al. [28] reported an association between the *DRD2* rs1076560 SNP and cocaine use, in EA but not the AA. The overall odds ratio of 1.94 in population (*n* = 214) was attributed to an artefact arising from the small sample size that was studied [9]. In a more recent study, Clark et al. [9] replicated the Moyer et al. [28] study by increasing sample size. The *DRD2* rs1076560 SNP was found to be associated with opioid use disorder in the two populations examined in this subsequent study (EA: *p *= 0.02, AA: *p *= 0.03), but not cocaine use (EA: *p *= 0.23, AA: *p *= 0.19). The MAF of the *DRD2* rs1076560 SNP, the “A allele”, was found to be higher in the cases when compared to controls in both populations: EA at 17.0% versus 14.0%, respectively, and in AA at 12.0% versus 9.0%, respectively [9].

The different association outcomes between the studies may account for the differences in the substance of use or the pattern of use in the cohorts that were studied. Table 1 summarises the type of substances in each study, which included alcohol, opioid, cocaine, and poly-substance use. Stratifying these studies based on the type or pattern of substance used is important to identify more specific genetic risk variants [31]. Iacono et al. [32] suggested that specific substances influenced the nature of the genes that are involved in the pharmacodynamics and pharmacokinetics of that substance [31, 32]. However, it is a challenge to stratify patients according to substance of use, as often there is no single substance that is used by patients and there are overlaps between the substances are used. Clark et al. [9] investigated associations with a single substance. However, their study was plagued with difficulties related to overlap between different types of the used substances [9]. In addition, the differences in the genetic architecture between populations could dictate whether a variant is associated or not (Table 1). For example, the association between *DRD2* rs1076560 SNP and alcohol use was statistically significant (*p* = 0.03, OR = 1.30) in a Japanese population [29]. However, the same SNP was not statistically significant in the Polish patients with alcohol use disorder (*p* = 0.14, OR = 1.30) [33]. Even though the findings of Malecka et al. [33] were not significant, the MAF “A allele” in the group of alcohol users was higher in the cases (21.0%) than in the controls (17.0%). In contrast, the MAF “A allele” in this UAE study and the AA group in Moyer et al. [28] were opposite, where the MAF “A allele” was higher in controls than in cases (Table 1).

This study found no significant genetic association between the *OPRM1* rs1799971 SNP (*p *= 0.12) with SUD among patients from the UAE population. The association between the *OPRM1* rs1799971 SNP and various phenotypes of SUD has been studied and includes being a risk factor to different types of substances of use including tobacco consumption [34], alcohol use and sensitivity [13, 19, 34, 35], and opioid use [16, 36]. Other studies looked into inducing clinical symptoms or mediating responses to therapeutic treatment [11, 17, 37, 38]. The association between the *OPRM1* rs1799971 SNP and SUD failed to reach statistical significance in our study (*p *= 0.12) and in a previous study by Enabah et al. [22] (*p *= 0.54). In addition, the MAF of the *OPRM1* rs1799971 SNP or “G allele” in our study (case = 15.4%, control = 18.9%) was distributed in a similar pattern to Enabah et al. [22] (case = 5.2%, control = 7.0%), where the MAF “G allele” was lower in cases than in the controls. However, by combining the two cohorts, as shown in Table 3, the increase in numbers resulted in a significant association between the *OPRM1* rs1799971 SNP and substance use. This suggests that a larger population size in future studies is required. The *OPRM1* rs1799971 SNP association varies and appears to depend on the study population and the nature of the substance of use. For example, Chen et al. [35] examined the association between the *OPRM1* rs1799971 SNP and alcohol use disorder in two different populations (Asian and Caucasian) in a meta-analysis study. They reported an association with the *OPRM1* rs1799971 SNP in Asians (*p *≤ 0.001) but not in Caucasian (*p *= 0.76). Other studies have reported a lack of association with alcohol use [24] and with heroin and/or cocaine use [16, 23]. Since the *OPRM1* rs1799971 SNP has been widely studied in different populations, we focused on compiling data based on the Arab studies (Table 2). Another Arab study by Al-Eitan et al. [21] investigated the role of *OPRM1* variants including the rs1799971 SNP on the outcomes of therapeutic treatment for opioids. This association between *OPRM1* rs1799971 SNP and the possibility of an increased chance of relapse in patients undergoing Naltrexone treatment for opioid use disorder in Jordanian patients was not significant (*p *= 0.55). The variability of the findings from the range of studies conducted to date highlights some contribution by the *OPRM1* rs1799971 SNP. However, the variability in associations found to date requires further study to understand the contribution of this SNP.

This study is the first to examine if there is any association between combined genotypes of two genes (*DRD2* rs1076560 SNP and *OPRM1* rs1799971 SNP) and the susceptibility to SUD in patients of Arabian ancestry. There was no significant association found between the combined genotype frequencies of the two SNPs and disease in this case–control study (cases *p* = 0.88 and controls *p* = 0.23). Although some studies support the combined effect of variants of these two genes (*DRD2* and *OPRM1*), the exact mechanism remains elusive. This may suggest the involvement of other genetic variants within or in the vicinity of the *DRD2* and *OPRM1* genes. For example, Zhang et al. [10] examined 13 SNPs in the *OPRM1* gene using haplotype analysis in an association study involving substance use patients from two populations: European and Russian ancestries [10]. They reported the involvement of the intronic variants of *OPRM1* (rs511435, rs534731, rs3823010, rs2075572, and rs609148) in increasing risk to SUD. Some of these SNPs were located in linkage disequilibrium (LD) with *OPRM1* rs1799971 SNP and others have been postulated to be involved in transcription regulation or alternative gene splicing. The findings in Zhang et al. [10] highlighted the limitation of selecting a single SNP of a candidate gene to examine the genetic association with SUD.

Interaction between the *DRD2* and *OPRM1* genes with other genes has been examined across different substances of use. For instance, Lechner et al. [39] examined the combined effect of the *OPRM1* rs1799971 SNP and the *DRD4* exon 3 VNTR variants on cigarette craving after alcohol consumption. The study reported that the presence of the G allele is associated with an increase in cigarette craving after alcohol consumption. However, no significant association between to the exon 3 VNTR variants of the *DRD4* were found with the condition [39]. In addition, Sullivan et al. [40] reported a gene–gene interaction between the dopamine receptors gene (*DRD2*) and the dopamine transporter gene (*DAT*) in cocaine users. The interaction between the regulatory variant of *DRD2* (rs2283265) and dopamine transporters gene altered *DAT* protein activity, supporting the possibility that variants being a risk factors for cocaine use [40].

## Conclusion

This study provides insights into two major genes that are thought to be risk factors of substance use. Specifically, the *DRD2* rs1076560 SNP and the *OPRM1* rs1799971 SNP were studied in substance use patients from the UAE population. No significant association between the *DRD2* rs1076560 SNP, the *OPRM1* rs1799971 SNP, and the combined genotype of the two SNPs and SUD was identified in this cohort. Nevertheless, future studies must consider stratification of the disease phenotype to assess possible association with *DRD2* and *OPRM1*. In addition, the findings of this study might suggest the involvement of other variants or genes in the mechanism of the disorder. Haplotype analyses for *DRD2* and *OPRM1* variants can be considered in future studies to evaluate the interaction of variants on each gene. Alternatively, genome-wide association study (GWAS) could be more objective strategy, since it does not rely on any previous conclusions from other populations.

## Abbreviations

SUD: substance use disorder
UAE: United Arab Emirates
NRC: National Rehabilitation Center
EFR: Emirates Family Registry
DSM-5: Diagnostic and Statistical Manual_5
SNP: single nucleotide polymorphism
QC: quality control
HWE: Hardy Weinberg equilibrium
AA: African American
EA: European American
VNTR: variable number of tandem repeats
